# Identification of COS markers specific for *Thinopyrum elongatum* chromosomes preliminary revealed high level of macrosyntenic relationship between the wheat and *Th*. *elongatum* genomes

**DOI:** 10.1371/journal.pone.0208840

**Published:** 2018-12-12

**Authors:** Eszter Gaál, Miroslav Valárik, István Molnár, András Farkas, Gabriella Linc

**Affiliations:** 1 Agricultural Institute, Centre for Agricultural Research, Hungarian Academy of Sciences, Martonvásár, Hungary; 2 Institute of Experimental Botany, Centre of the Region Haná for Biotechnological and Agricultural Research, Olomouc, Czech Republic; Institute of Genetics and Developmental Biology Chinese Academy of Sciences, CHINA

## Abstract

*Thinopyrum elongatum* (Host) D.R. Dewey has served as an important gene source for wheat breeding improvement for many years. The exact characterization of its chromosomes is important for the detailed analysis of prebreeding materials produced with this species. The major aim of this study was to identify and characterize new molecular markers to be used for the rapid analysis of E genome chromatin in wheat background. Sixty of the 169 conserved orthologous set (COS) markers tested on diverse wheat-*Th*. *elongatum* disomic/ditelosomic addition lines were assigned to various *Th*. *elongatum* chromosomes and will be used for marker-assisted selection. The macrosyntenic relationship between the wheat and *Th*. *elongatum* genomes was investigated using EST sequences. Several rearrangements were revealed in homoeologous chromosome groups 2, 5, 6 and 7, while chromosomes 1 and 4 were conserved. Molecular cytogenetic and marker analysis showed the presence of rearranged chromosome involved in 6ES and 2EL arms in the 6E disomic addition line. The selected chromosome arm-specific COS markers will make it possible to identify gene introgressions in breeding programmes and will also be useful in the development of new chromosome-specific markers, evolutionary analysis and gene mapping.

## Introduction

Wild relatives of bread wheat (*Triticum aestivum* L.) are important sources of agriculturally useful traits and alleles for wheat improvement. Wild gene variants can be transferred by interspecific or intergeneric hybridization in order to increase the allelic diversity of wheat [1]. Species from the *Thinopyrum* genus have long been used as genetic sources of salinity, drought and low temperature tolerance [2,3] and high grain micronutrient and protein content [4–6]. Several genes for resistance to leaf and stem rusts, wheat streak mosaic virus, barley yellow dwarf virus, Fusarium head blight and *Cephalosporium* stripe disease have already been identified and used in wheat breeding programmes [7–12].

*Thinopyrum elongatum* (Host) D.R. Dewey (= *Agropyron elongatum* (Host) P. Beauvois, *Elytrigia elongata* (Host) Nevski, *Lophopyrum elongatum* (Host) A. Löve, 2n = 2x = 14, EE) belongs to the tertiary gene pool of wheat. *Th*. *elongatum* can easily be crossed with wheat, and several resistance genes for rust resistance (*Lr19*, *Lr24*, *Lr29*, *Sr24*, *Sr25*, *Sr26*, *Sr43*) and wheat streak mosaic virus have been transferred into wheat [1]. It was proved that the substitution of chromosomes 2E and 3E into a Chinese Spring (CS) background resulted in resistance to cereal yellow dwarf virus, while chromosomes 1E and 6E provided resistance to *Septoria tritici* blotch [13]. *Th*. *elongatum* is also considered as a promising gene source for salt tolerance [3]. By investigating the salt tolerance of wheat-*Th*. *elongatum* amphiploid and chromosome substitution lines, Omielan et al. [14] found that chromosome 3E has a positive effect on grain yield under stress conditions relative to the wheat parent. Under optimal conditions, a positive effect on the grain yield was also found in a 7DL.7Ag translocation line containing the *Lr19* gene [15,16]. *Th*. *elongatum* has been reported to be a source of perennial growth habit, as found for the Chinese Spring-*Th*. *elongatum* 4E disomic addition line by Lammer et. al. [17], who reported that the 4E addition line was able to produce a second crop of seed under favourable conditions.

The chromosome-mediated transfer of *Thinopyrum* genes is only successful if the introgressed alien chromosome segment has the same gene content as that of the corresponding wheat chromatin, whereby the alien segment is able to compensate for the loss of wheat chromatin. However, the homoeologous relationships between wheat and alien chromosomes could have been destroyed by evolutionary genome rearrangements formed in wheat and alien species since their evolutionary divergence [18,19]. Compensating wheat-alien translocations are generally the consequence of meiotic recombination between the homoeologous chromosomes of wheat and its wild crossing partner. However, differences in the genome structure of wheat and the wild gene source species may restrict the meiotic pairing between wheat and alien chromosomes even in the absence of the *Ph1* (*Pairing homoeologous 1*) locus, the major component of the genetic system ensuring homologous chromosome pairing in wheat [20]. For the efficient production of compensating translocations between wheat and *Th*. *elongatum* it is important to analyze and understand the homoeology between the genomes of wheat and *Th*. *elongatum*.

In wheat introgression-breeding programmes it is essential to identify the alien chromatin in wheat background, which determines the efficiency of chromosome-mediated gene transfer. Molecular cytogenetic methods are widely used to detect and identify alien chromatin in wheat [21]. *In situ* hybridization using labelled total genomic DNA as probes (genomic *in situ* hybridization, GISH) allows alien chromosomes and chromosome segments to be visualized [22,23]. The individual chromosomes of a species can be identified by the hybridization pattern of repetitive DNA probes after fluorescence *in situ* hybridization (FISH) [24,25]. Although *Th*. *elongatum* has long been used in wheat breeding programmes its detailed FISH karyotype with the probes pTa71, pSc119.2 and Afa family was only reported a few years ago [26]. Cytogenetic methods are powerful techniques, but they are less efficient for identifying small introgressions or screening large prebreeding populations. The low-throughput of cytogenetic selection methods is one of the main limitations hampering the identification of wheat-*Th*. *elongatum* introgression lines.

The application of molecular markers to select desirable wheat-*Th*. *elongatum* introgressions would be a better option because of their high throughput [27]. However, in the case of *Th*. *elongatum* only a few molecular markers are available, a fact that limits the high-throughput marker-assisted selection of introgression lines and also slows down the development of high density genetic and physical maps, the mapping of favourable agronomic traits and the map-based positional cloning of genes in *Th*. *elongatum*.

In the last decade several molecular markers were established for *Th*. *elongatum*, such as SSR markers [28], EST-SSR markers [29,30], RAPD- and ISSR-based SCAR markers [31] and CAPS markers [32]. Specific length amplified fragment sequencing (SLAF) markers are also available, especially for the 7E chromosome [33]. Recently Lou et al. [34] reported the high‐throughput mining of E‐genome‐specific SNPs potentially suitable for the selection of *Th*. *elongatum* introgressions in hexaploid wheat. However, the use of SNP markers is labour-intensive and requires detailed sequence information and needs.

In the last decade, comparative genomic studies between wheat and rice or *Brachypodium* identified a set of conserved orthologous genes referred to as conserved orthologous set (COS) markers [35,36]. As the primers were designed to overlap the exon-intron boundaries of conserved genes, COS markers are potentially highly polymorphic, because they span the introns, which have a more frequent polymorphisms than exons [37]. These markers are specific for the orthologous regions, thus allowing the comparison of the chromosome structure in related species such as rice, wheat, maize, sorghum and barley [38]. It was shown that COS markers are highly transferable between species in the *Triticeae*, such as *Ae*. *peregrina* and *Ae*. *ventricosa* [36,39]. Molnár et al. [40,41] used COS markers to compare the genome structure of wheat and of *Aegilops* species with U, M, S and C genomes at the chromosome level.

Recently COS markers were successfully used to investigate phylogenetic relationships between three diploid wheatgrass species, *Agropyron cristatum* (L.) Beauv., *Thinopyrum bessarabicum* (Savul.&Rayss) A. Löve and *Pseudoroegneria spicata* (Pursh) A. Löve [42]. Therefore, COS markers have potential for the comparison of chromosome-level orthologous relationships between the E genome and the A, B and D genomes of wheat. On the other hand, to date no COS markers have been assigned to the E-genome chromosomes of *Th*. *elongatum*, which hampers the comparative genome analysis of this important gene source species relative to wheat.

The main goal of the present study was to make chromosome-mediated gene transfer from *Thinopyrum* more effective in wheat prebreeding programmes by identifying suitable COS markers. To achieve this goal, wheat-specific COS markers were assigned to the E-genome chromosomes of *Th*. *elongatum* using PCR with DNA from a wheat-*Thinopyrum elongatum* amphiploid, a complete set of wheat-*Th*. *elongatum* disomic addition lines, and 12 ditelosomic addition lines. A further aim was to study orthologous relationships between the chromosomes of *Thinopyrum elongatum* and bread wheat, using a sequence similarity search between the source ESTs of the assigned COS markers and the reference sequences of wheat chromosomes.

## Materials and methods

### Plant materials

A complete set of Chinese Spring-*Th*. *elongatum* disomic addition lines (1E, 2E, 3E, 4E, 5E, 6E, 7E), 12 ditelosomic addition lines (1ES, 1EL, 2ES, 2EL, 3ES, 3EL, 4ES, 5ES, 5EL, 6ES, 7ES, 7EL) and a Chinese Spring-*Th*. *elongatum* amphiploid line (Amp) containing the whole set of *Th*. *elongatum* chromosomes were developed by Dvorak and Knott [43] and kindly provided by Prof. Jan Dvorak for the present study. The parental wheat (*Triticum aestivum* L.) genotype Chinese Spring (CS) was used as a control. For *in situ* hybridization the DNA of accession of *Th*. *elongatum* MvGB1963 (PI578686) and the wheat genotype CS were isolated and labelled. All the material is maintained in the Cereal Gene Bank, Agricultural Institute, Centre for Agricultural Research, Hungarian Academy of Sciences, Martonvásár, Hungary.

### In situ hybridization

*In situ* hybridization was performed to confirm the chromosomal constitution of the amphiploid and the addition lines. Chromosome preparation and GISH were carried out according to Linc et al. [44]. The *Th*. *elongatum* total genomic DNA was labelled with biotin-11-dUTP by nick translation (Biotin-Nick Translation Mix; Roche). Unlabelled wheat DNA was added as a competitor at 50× the probe amount in order to block common sequences. FISH was performed using the repetitive DNA probes Afa family, pSc119.2 and pTa71. The repetitive DNA sequences Afa family [45] and pSc119.2 [46] were amplified and labelled with biotin-11-dUTP (Roche) and digoxigenin-16-dUTP (Roche) by PCR. The 18S-5.8S-26S rDNA clone pTa71 [47] was labelled with 50% biotin-11-dUTP (Roche) and 50% digoxigenin-16-dUTP (Roche) by nick translation. Biotin and digoxigenin were detected by streptavidin-FITC (Roche) and anti-digoxigenin-rhodamine Fab fragments (Roche). The chromosomes were counterstained with 2 μg/ml DAPI (4’-6-diamino-2-phenylindole) and mounted in antifade solution. A Zeiss AxioImager M2 epifluorescence microscope equipped with filter sets for detecting DAPI, FITC and Rhodamine signals was used to document the fluorescence signals. Images were captured with a Zeiss AxioCam MRm CCD camera and processed with Zeiss AxioVision 4.8.2. software.

### COS marker analysis

Total genomic DNA was extracted from fresh young leaves of the CS-*Th*. *elongatum* disomic and ditelosomic addition lines and the Th. elongatum-CS amphiploid and the parental wheat CS using a QuickGene-Mini80 device (FujiFilm, Osaka, Japan) with a QuickGene DNA tissue kit (FujiFilm) according to the manufacturer’s instructions.

A total of 169 markers (149 COS and 20 InDel markers) (whose primer sequences and PCR conditions are given in S1 Table) potentially covering wheat homoeologous groups I-VII were chosen from publicly available COS marker collections [35,36].

The PCR reactions were performed in a total reaction volume of 12 μl, containing 1× PerfectTaq Plus PCR Buffer (5 Prime GmbH, Hamburg, Germany), 0.4 μM primers and 50 ng genomic DNA as template, as described by Molnár et al. [41], using a touchdown reaction profile: 94°C (2 min), 10 cycles of [94°C (0.5 min), Ta + 5°C (0.5 min), decreasing in 0.5°C increments for every subsequent set of cycles, 72°C (1 min)], 30 cycles of 94°C (0.5 min), Ta°C (0.5 min), 72°C (1 min), hold at 72°C (2 min) then at 10°C in an Eppendorf Mastercycler (Eppendorf, Hamburg, Germany).

The PCR amplicons were separated with a Fragment Analyzer^™^ Automated CE System equipped with a 96-Capillary Array Cartridge (effective length 33 cm) (Advanced Analytical Technologies, Ames, USA) and the results were analysed and visualized with PROSize v2.0 software (Advanced Analytical Technologies, USA), as summarized in S2 Table.

### Development of InDel markers

In order to enrich the 2DL and 6DL chromosome arms with markers and investigate wheat- *Thinopyrum elongatum* homeologous relationships between the long arms of wheat and *Thinopyrum*, we used InDel markers previously designed for *Aegilops umbelluata*. Twelve and eight wheat ESTs specific for the long arms of chromosome 2D and 6D, respectively, were selected from the (https://wheat.pw.usda.gov/cgi-bin/westsql/map_locus.cgi) database. The wheat EST sequences were aligned to the genomic sequences of the *Aegilops umbellulata* AE740/03 [48] using BLASTn with the program Ragged Genes Genome Explorer (version 2.2.24) which is a graphic interface for NCBI’s BLAST command line tool [49]. BLASTn hits that met certain criteria (≥7–8 bp InDel polymorphism and ≥70% homology between *Ae*. *umbellulata* and wheat sequences) were selected and a pairwise alignment of the selected ESTs and *Aegilops* contigs was carried out with UGENE software (v.1.27.0) to identify InDel regions between wheat and *Aegilops* and to design primers to amplify the polymorphic regions. Primers were designed to amplify the InDel regions with the Primer3 tool integrated into the UGENE software. Primers were synthesized by Integrated DNA Technologies (Coralville, Iowa, USA).

### Sequence analysis

To assess chromosomal synteny between bread wheat and diploid *Th*. *elongatum* the source EST sequences of the COS markers were aligned with the Chinese Spring reference sequence v1.0 [50] using BLASTn algorithm [51]. Only alignments with E-values smaller than 2.8e^-08^, identity > 82% and alignment length > 100bp were considered as significant (S3 Table). Positions of the first basepair (bp) of the best hit in the wheat pseudomolecule was used as physical positions of each COS marker. The lengths (in bp) of the wheat pseudomolecules and the start genomic positions of the ESTs were converted to pixels and physical maps of the COS markers were designed using custom-made software (http://geneticmap.herokuapp.com/). To visualize the wheat-*Th*. *elongatum* syntenic relationships, the markers on the wheat physical map were colour-coded to show the chromosomal locations in *Th*. *elongatum*.

## Results

### In situ hybridization

**Fig 1:**
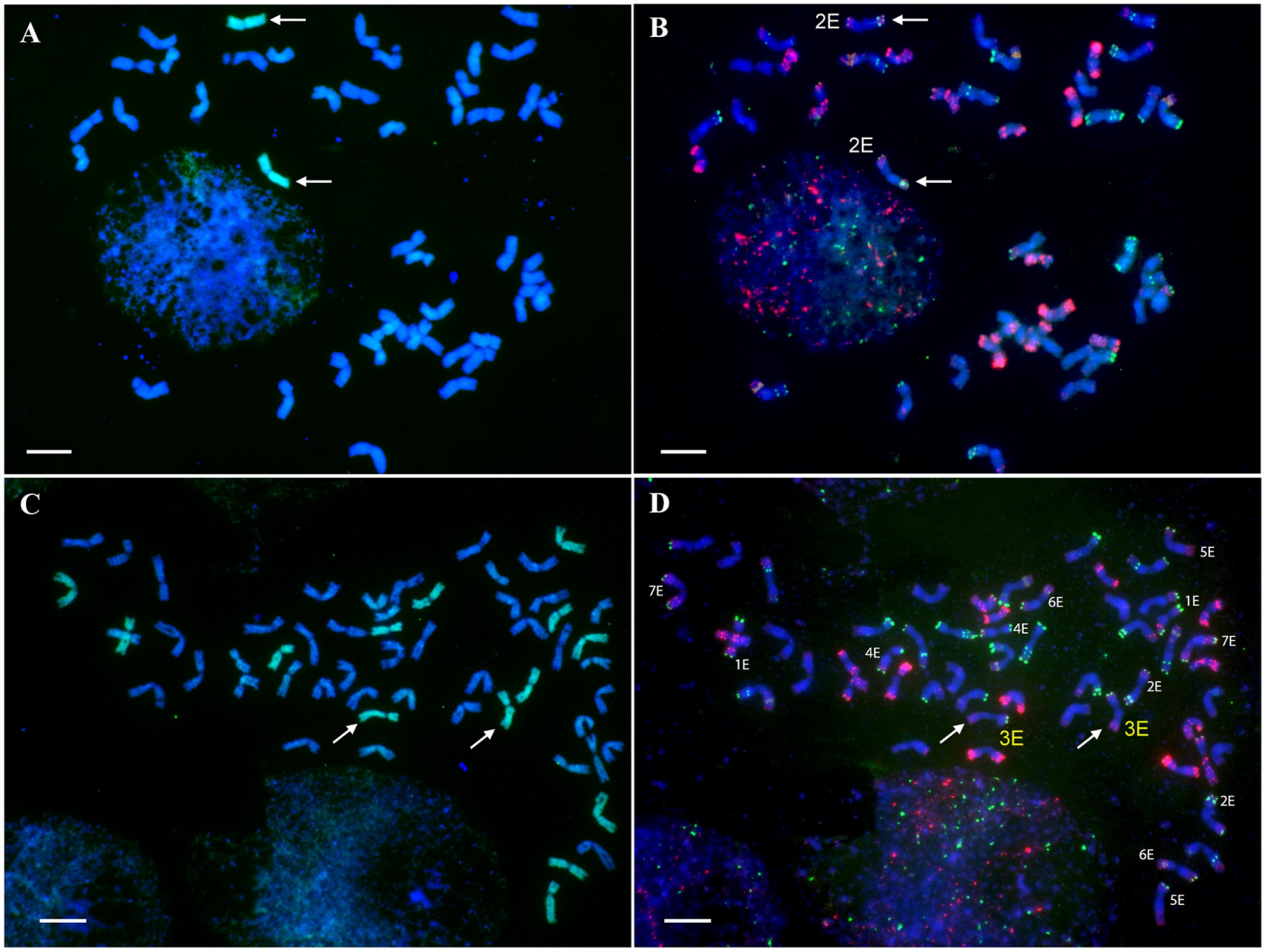
Molecular cytogenetic analysis of the wheat-*Th*. *elongatum* addition lines. Example of presence and identity verification of the wheat- *Th*. *elongatum* addition lines. A mitotic metaphase cell of the 2E disomic addition line after genomic *in situ* hybridization (GISH) (A) and fluorescence *in situ* hybridization (FISH) (B). In the wheat-*Th*. *elongatum* amphiploid (C and D), GISH (C) and FISH (D) detected the complete set of E-genome chromosomes, including chromosome 3E. In the GISH images the E genome was visualized in green, while in the FISH images the repetitive DNA probes pSc119.2, Afa family and pTa71 were visualized in green, red and yellow, respectively. Chromatin was nonspecifically stained with DAPI (blue). Scale bar = 10μm.

The wheat-*Th*. *elongatum* disomic and ditelosomic addition lines were verified for presence of E-genome chromosomes using GISH. The identity of the E genome chromosomes and their arms was verified by FISH with the pSc119.2, Afa and pTa71 probes [26]. The cytogenetic analysis confirmed the presence of 1E, 2E, 4E, 5E and 7E chromosomes in the disomic and ditelosomic addition lines (Fig 1A and 1B; S1–S5 Figs). However, the 3E addition line was not confirmed and thus it was omitted from further analysis. In order to assign markers to chromosome 3E, the wheat-*Th*. *elongatum* amphiploid line was included in the experiments (Fig 1C and 1D).

**Fig 2:**
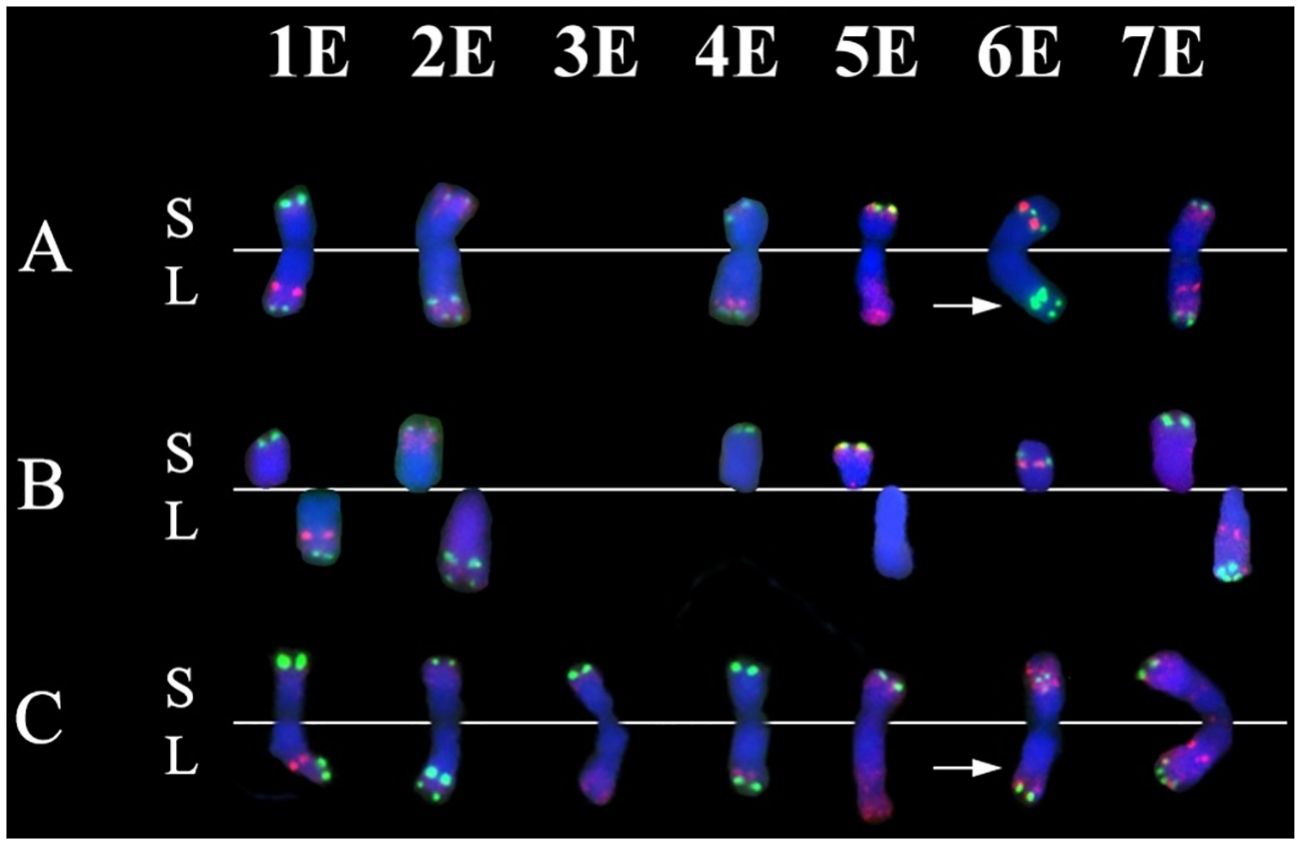
The comparison of the FISH karyotypes of *Th*. *elongatum* chromosomes 1E-7E identified in the genetic stocks used in this study. Chromosomes 1E-7E (except 3E) from wheat-*Th*. *elongatum* disomic addition lines (A), E genome chromosome arms from wheat-*Th*. *elongatum* ditelosomic addition lines (B) and chromosomes 1E-7E (except 3E) from the wheat-*Th*. *elongatum* amphiploid line (C). Repetitive DNA probes pSc119.2, Afa family and pTa71 were visualized in green, red and yellow, respectively. The chromatin was nonspecifically stained with DAPI (blue). A horizontal white line indicates the position of the centromere. Scale bar = 10μm.

When the hybridization pattern of the 6E chromosome detected in the addition line was compared to those of the amphiploid line (Fig 2), it was found that the FISH pattern of the 6E long arm in the addition line was highly similar to the long arm of 2E chromosome present in the amphiploid line, in the 2E addition line or in the 2EL ditelosomic line (Fig 1; S3 Fig). On the other hand, the long arm of chromosome 6E of the amphiploid, with the typical hybridisation pattern, was not found in any addition lines. Therefore we concluded that the wheat-*Th*. *elongatum* addition line contains a rearranged *Th*. *elongatum* chromosome involving the chromosome arms 6ES and 2EL.

### COS marker analysis

**Fig 3:**
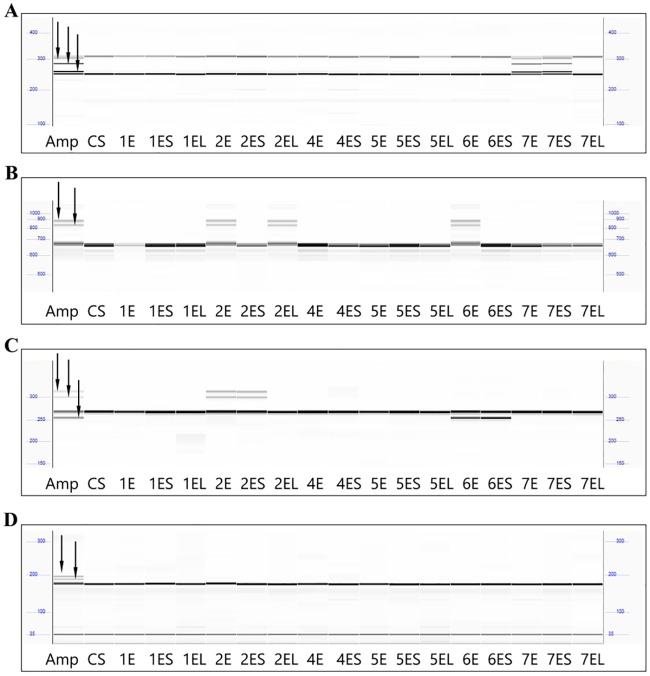
Fragment profiles of the COS markers on the CS- *Th*. *elongatum* addition lines. Representative digital gel images for the assignment of markers to *Thinopyrum elongatum* chromosomes using the CS-*Th*. *elongatum* amphiploid (Amp), the parental wheat genotype Chinese Spring (CS) and the CS-*Th*. *elongatum* chromosome- (1E-7E) or chromosome arm (1–7 ES or EL) addition lines. (A) Polymorphic bands specific for one *Th*. *elongatum* chromosome as shown for marker *TR335*. (B) Polymorphic bands specific for several E-genome chromosomes shown for the marker *c746642*. (C) Different polymorphic bands specific for different E-genome chromosomes shown for the marker *TR430*. (D) Polymorphic bands assumed to be specific for 3E chromosome as shown for the marker *TR62*. Digital gel image were produced using PROSize 2.0 Software.

A total of 169 markers (149 COS and 20 InDel) specific for wheat homoeologous groups 1–7 were used for the present experiments to assign specific markers for all seven E genome chromosomes. Among the 169 markers, 156 (92.3%) showed PCR products in at least one of the 18 genotypes analysed, while 13 markers (7.7%) amplified no products (S2 Table). The 156 markers resulted in 400 PCR products (1–7 PCR products/marker/genotype; mean: 2.81 products/marker), of which 116 (29.0%) showed size polymorphism (≥ 4bp) between the amphiploid line and the parental wheat genotype CS (Fig 3), while 284 bands (71.0%) showed no polymorphism.

The 116 polymorphic amplicons originated from 60 markers. The main polymorphic amplicons of these 60 markers are presented in Table 1. Thirty (50%) of these 60 markers could be assigned to a single E-genome chromosome arm on the basis of chromosome or chromosome arm addition lines (1ES:1; 1EL:3; 2ES:3; 4ES:2; 4EL:6; 5ES:1; 5EL:3; 6ES:7; 7ES:3; 7EL:1) (Fig 3A; Table 1; S2 Table).

**Table 1:** The list of chromosome-specific COS markers and their main polymorphic products in *Thinopyrum elongatum* (For more details see S2 Table).

Marker	Chromosomal location in wheat	Chromosomal location in *Th*. *elongatum*	Amplicon (bp)
*c737520*	1AS, 1BS, 1DS	1ES	377
*c757212*	1AL, 1BL, 1DL	1EL	262
*c744533*	1AL, 1BL, 1DL	1EL	245
*TR574*	1AL, 1BL, 1DL	1EL	238
*2R*	2AS, 2BS, 2DS	2ES	297
*TR416*	2AS, 2BS, 2DS	2ES	408
*TR725*	2AS, 2BS, 2DS	2ES	414
TR430*	2AS, 2BL, 2DS	2ES	315
	2AS, 2BL, 2DS	6ES	255
TR636*	2AL, 2BL, 2DL	2EL,6EL	248
	2AL, 2BL, 2DL	6ES	328
*2N*	2AL, 2BL, 2DL	2EL,6EL	673
*c744070*	2AL, 2BL, 2DL	2EL,6EL	245
*TR146*	2AL, 2BL, 2DL	2EL,6EL	319
*c746642*	2AL, 2BL, 2DL	2EL,6EL	892
*BE424990*	2DL	2EL,6EL	355
*BE443185*	2DL	2EL,6EL	456
*BE495372*	2DL	2EL,6EL	519
*TR62*	3AL, 3BL, 3DL	3E^#^	196
*c756059*	3AS, 3BS, 3DS	3E^#^	492
*c755442*	3AS, 3BS, 3DS	3E^#^	868
*TR63*	3AL, 3BL, 3DL	3E^#^	564
*c752685*	3AS, 3BS, 3DS	3E^#^	650
*c767527*	3AL, 3BL, 3DL	3E^#^	375
*TR67*	3AL, 3BL, 3DL	3E^#^	338
*TR85*	3AS, 3BS, 3DS	3E^#^	244
*TR4*	3AS, 3BS, 3DS	3E^#^	321
*c765452*	4AS, 4BL, 4DL	4ES	389
*TR131*	4AL, 4BS, 4DS	4ES	363
*4C*	4AL, 4BS, 4DS	4EL	439
*c770094*	4AS, 4BS, 4DL	4EL	461
*c760004*	4AL, 4BS, 4DS	4EL	997
*c771467*	4AL, 4BS, 4DS	4EL	291
*TR759*	5AL, 5BL, 5DL	4EL	349
*TR471*	5AL, 5BL, 5DL	5ES	340
*c764126*	5AL, 5BL, 5DL	5EL	275
*TR513*	5AL, 5BL, 5DL	5EL	352
*TR757*	5AL, 5BL, 5DL	5EL	220
c749645*	5AL, 5BL, 5DL	5EL,7ES	350
	5AL, 5BL, 5DL	5EL	311
	5AL, 5BL, 5DL	7ES	399
*TR451*	5AL, 5BL, 5DL	2EL,6EL	320
*TR90*	6AS, 6BS, 6DS	6ES	310
*TR91*	6AS, 6BS, 6DS	6ES	368
*TR88*	6AS, 6BS, 6DS	6ES	482
*c747871*	6AS, 6BS, 6DS	6ES	789
*TR92*	6AS, 6BS, 6DS	6ES	244
*BE498099*	6DS	6ES	371
*TR93*	6AS, 6BS, 6DS	6ES	502
*c741435*	6AL, 6BL, 6DL	4EL	507
*739776*	6AL, 6BL, 6DL	6EL^##^	390
*742079*	6AL, 6BL, 6DL	6EL^##^	405
*725983*	6AL, 6BL, 6DL	6EL^##^	164
*755465*	6AL, 6BL, 6DL	6EL^##^	790
*756643*	6AL, 6BL, 6DL	6EL^##^	366
*757249*	6AL, 6BL, 6DL	6EL^##^	1122
*760225*	6AL, 6BL, 6DL	6EL^##^	354
*765143*	6AL, 6BL, 6DL	6EL^##^	396
*BE518349*	6DL	6EL^##^	415
*7C*	7AS, 7BS, 7DS	7ES	448
*TR355*	7AS, 7BS, 7DS	7ES	252
*TR335*	7AS, 7BS, 7DS	7ES	260
TR151*	7AS, 7BS, 7DS	7ES	554
	7AS, 7BS, 7DS	7EL	207
*c753911*	7AL, 7BL, 7DL	7EL	166

^#^Amplicon produced only on CS-*Th*. *elongatum* amphiploid, so the putative location of the marker is 3E, since the source EST of the marker was w3 specific.

^##^Amplicon produced only on CS-*Th*. *elongatum* amphiploid, so the putative location of the marker is 6EL, since the source EST of the marker was specific for the long arm of w6.

*: Markers producing different polymorphic bands specific for different E-genome chromosomes (Fig 2C).

The nine markers specific for the long arm of wheat 6 (w6) chromosomes and produced polymorphic amplicons in the amphiploid, failed to produce amplicons in the wheat-*Th*. *elongatum* 6E addition line. We have to note, that the 7 markers specific for the short arm of w6 chromosomes were detected in the amphiploid and also in the 6E addition line suggesting the presence of 6ES and the absence of 6EL arms in the wheat-*Th*. *elongatum* 6E addition line. However, the 8 markers specific for the long arm of w2 chromosomes were mapped on the amphiploid, the 2E and 2EL additions and also on the 6E addition line (Fig 3B; Table 1) suggesting the presence of 2EL arm in the wheat-*Th*. *elongatum* 6E addition line. All of these results agreed well with the results of molecular cytogenetic analysis (Fig 2) and supporting our hypothesis that the E-genome chromosome present in the wheat-*Th elongatum* 6E addition line is rearranged chromosome involved in the chromosome arms 6ES and 2EL.

Nine out of the 60 markers specific for the w3 chromosomes could be mapped to the amphiploid but not to any of these chromosomes, suggesting these markers are located on the missing 3E chromosome (Fig 3D). Twelve out of the 60 markers (20.0%) could be detected on more than one *Th*. *elongatum* chromosome (Fig 3B and 3C). Eight out of these 12 markers the same-size amplicons were detected on different chromosomes (2EL and 6EL), as shown for marker *c746642* (Fig 3B), while the different-sized amplicons of the remaining four markers were specific for different E-genome chromosomes, as shown for TR430 (Fig 3C).

*Th*. *elongatum* chromosome-specific markers with a significant level (≥4bp) of length polymorphism between the parental wheat Chinese Spring and CS-*Thinopyrum elongatum* hybrid progenies were considered to be suitable for the marker-assisted selection of new wheat-*Thinopyrum* introgression lines in prebreeding programmes.

### Sequence analysis

**Fig 4:**
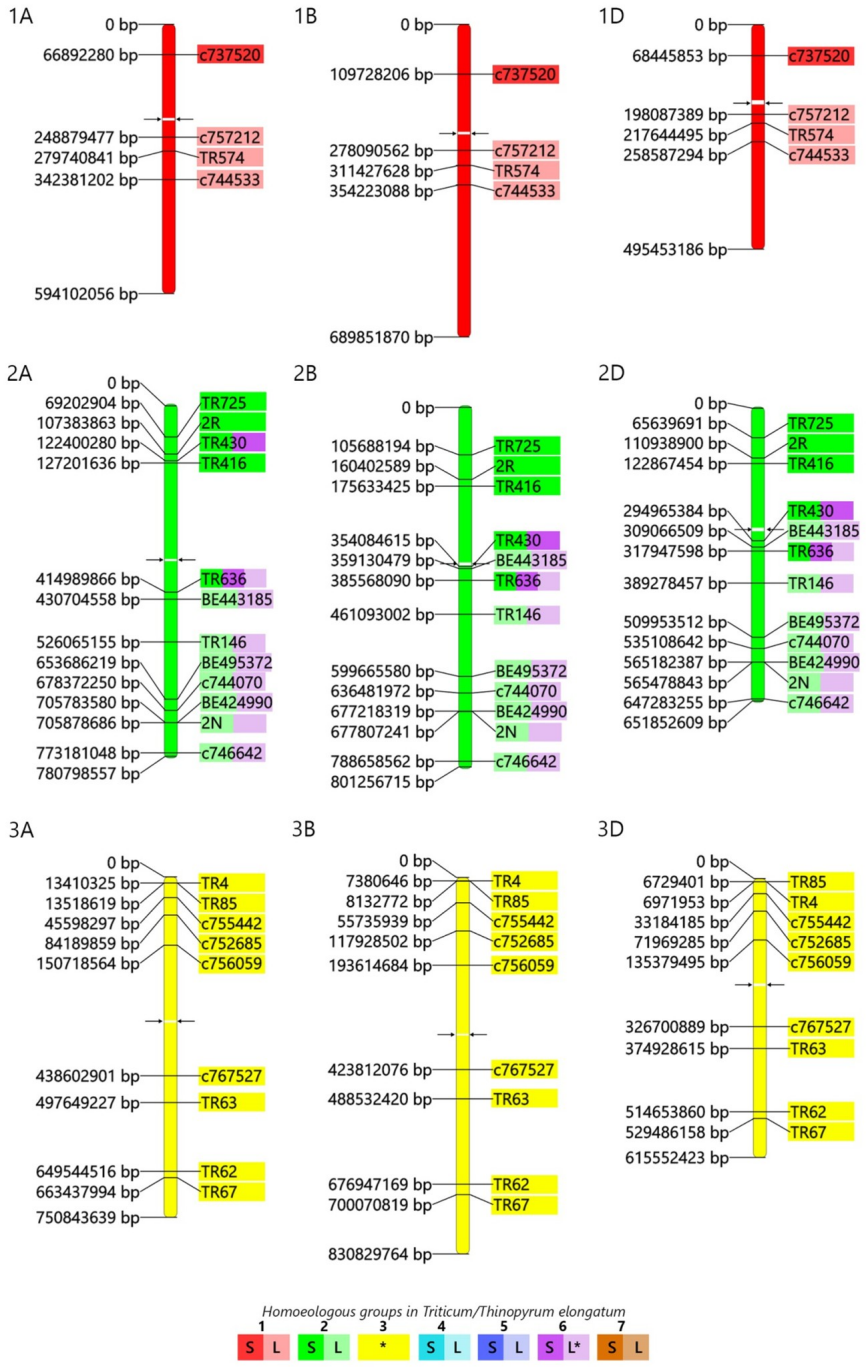
Wheat—*Th*. *elongatum* orthologous relationships. The physical map of wheat pseudomolecules shows the positions of COS markers (on the left) on wheat chromosome groups 1–3, while the marker positions on *Th*. *elongatum* chromosomes are visualized by the coloured marker names (on the right). The marker positions on the wheat chromosomes were obtained by a BLASTn search for the source ESTs of the markers against the IWGSC wheat pseudomolecules (in bp values) and the start positions of the best hits were used for the map design. To indicate the chromosomal location in *Th*. *elongatum* the marker names were coloured according to the PCR results on wheat-*Th*. *elongatum* genetic stocks. Full colour shows markers located on the short arm, while half tone colour indicates location on the long arm. Bicolour markers indicate duplicated loci. In the wheat chromosomes arrowheads indicate the position of the centromere. *: The chromosomal location in *Th*. *elongatum* determined by the use of wheat-*Th*. *elongatum* amphiploid.

**Fig 5:**
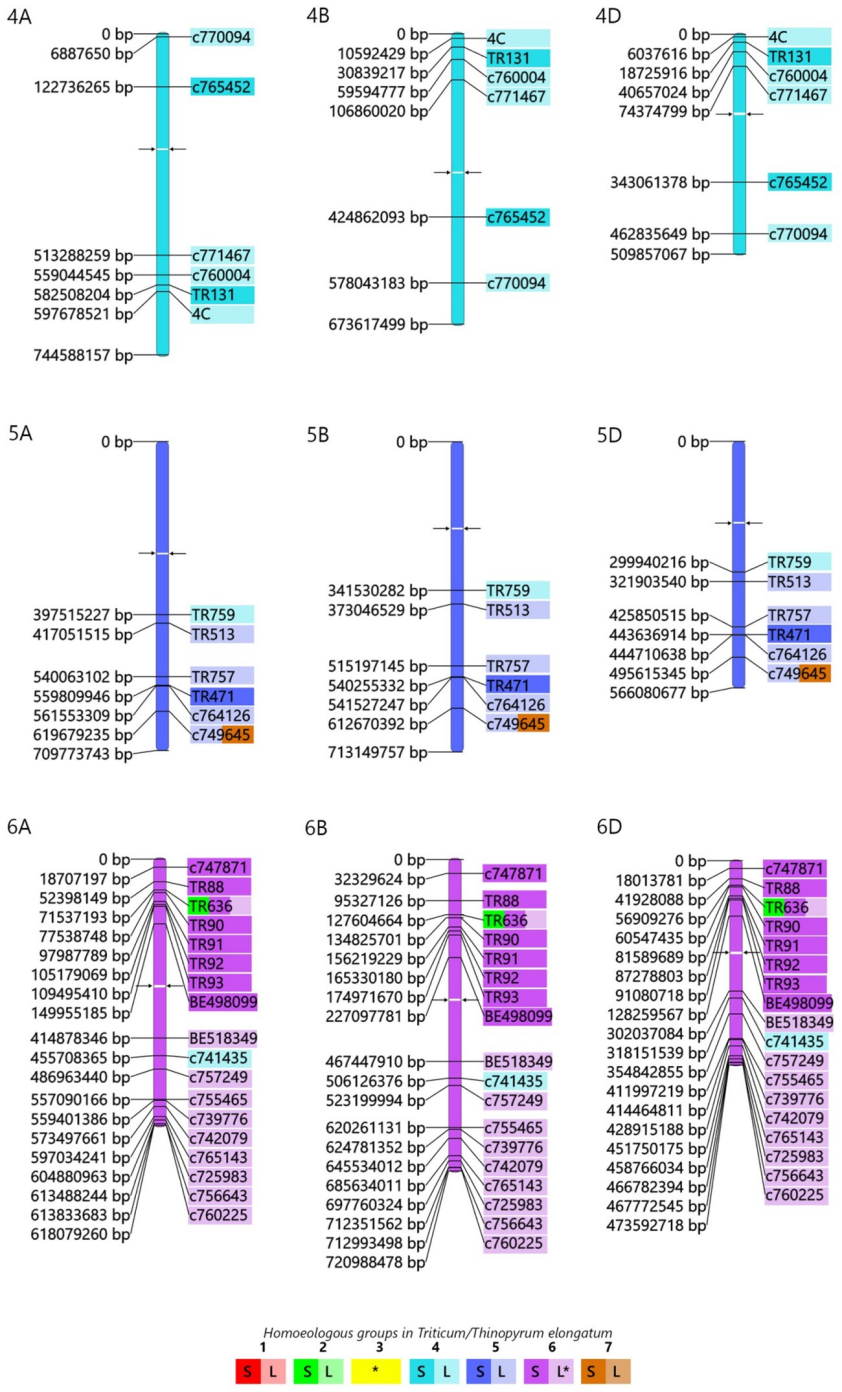
Wheat—*Th*. *elongatum* orthologous relationships. The physical map of wheat pseudomolecules shows the positions of COS markers (on the left) on wheat chromosome groups 4–6, while the marker positions on *Th*. *elongatum* chromosomes are visualized by the coloured marker names (on the right). The marker positions on the wheat chromosomes were obtained by a BLASTn search for the source ESTs of the markers against the IWGSC wheat pseudomolecules (in bp values) and the start positions of the best hits were used for the map design. To indicate the chromosomal location in *Th*. *elongatum* the marker names were coloured according to the PCR results on wheat-*Th*. *elongatum* genetic stocks. Full colour shows markers located on the short arm, while half tone colour indicates location on the long arm. Bicolour markers indicate duplicated loci. In the wheat chromosomes arrowheads indicate the position of the centromere. *: The chromosomal location in *Th*. *elongatum* determined by the use of wheat-*Th*. *elongatum* amphiploid.

**Fig 6:**
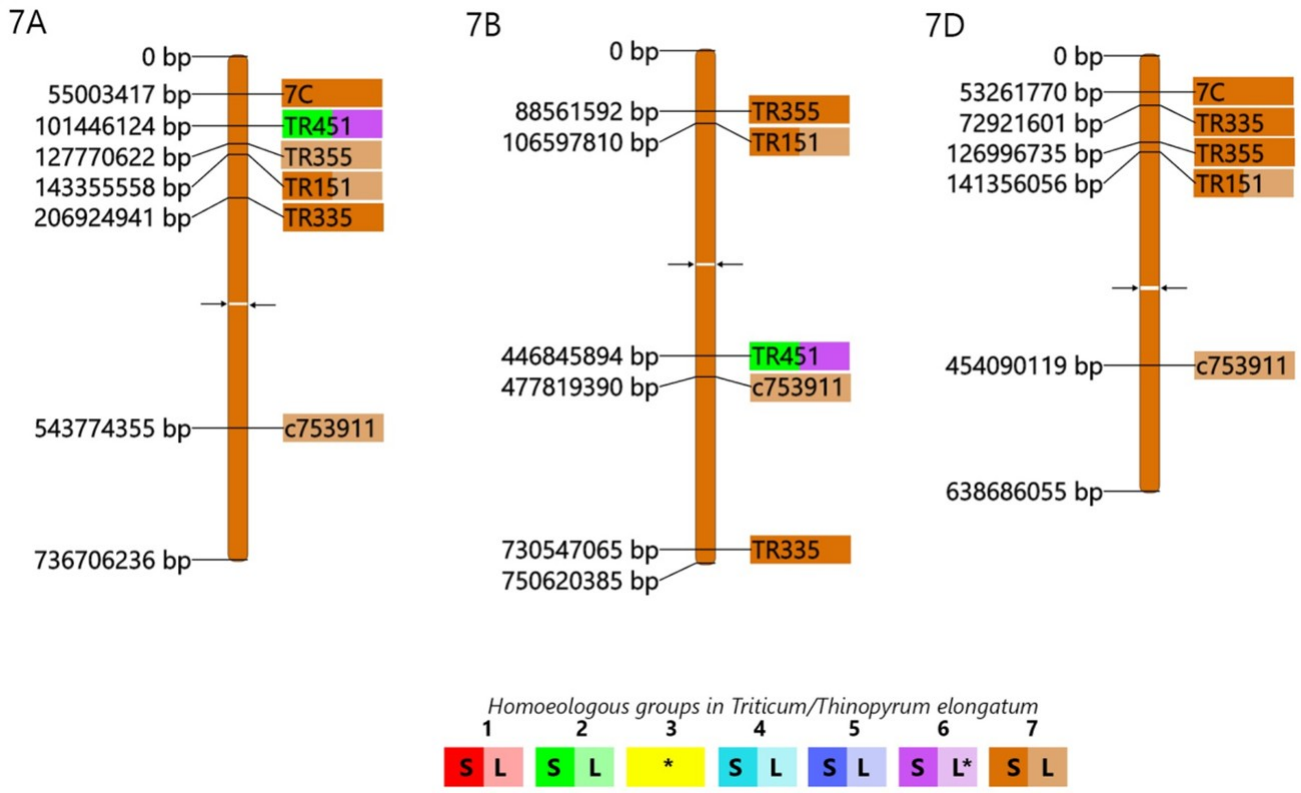
Wheat—*Th*. *elongatum* orthologous relationships. The physical map of wheat pseudomolecules shows the positions of COS markers (on the left) on wheat chromosome group 7, while the marker positions on *Th*. *elongatum* chromosomes are visualized by the coloured marker names (on the right). The marker positions on the wheat chromosomes were obtained by a BLASTn search for the source ESTs of the markers against the IWGSC wheat pseudomolecules (in bp values) and the start positions of the best hits were used for the map design. To indicate the chromosomal location in *Th*. *elongatum* the marker names were coloured according to the PCR results on wheat-*Th*. *elongatum* genetic stocks. Full colour shows markers located on the short arm, while half tone colour indicates location on the long arm. Bicolour markers indicate duplicated loci. In the wheat chromosomes arrowheads indicate the position of the centromere. *: The chromosomal location in *Th*. *elongatum* determined by the use of wheat-*Th*. *elongatum* amphiploid.

In order to compare the structure of the E genome of *Th*. *elongatum* with the A, B and D genomes of bread wheat, the source EST sequences of the 60 COS markers producing polymorphic amplicons on the E-genome chromosomes (Table 1) were aligned to the reference sequences of the wheat chromosomes (https://www.wheatgenome.org/). To do this, a BLASTn sequence similarity search was made for source EST sequences against the IWGSC wheat pseudomolecules (S3 Table). Based on the start positions of the aligned sequences of the best hits, a physical map was constructed for all the chromosomes of wheat homoeologous groups 1–7 (Figs 4–6).

The physical maps provide an overview, from the A-, or B- and D-genome perspective, of the wheat-*Thinopyrum elongatum* genome relationships at the resolution level of *Triticum*/*Thinopyrum* chromosomes (Figs 4–6). The coverage of wheat chromosome groups I, IV, V and VII with COS markers (4, 6, 5 and 5 markers/chromosome, respectively) was smaller than that of the remaining chromosome groups (II, III and VI with 12, 9 and 17 markers, respectively). Most of the markers were located on the same homoeologous group chromosomes in *Thinopyrum* as in wheat. It is important to note that 9 markers, assigned indirectly to chromosome 3E based on the presence of E-genome specific amplicons in the CS-*Th*. *elongatum* amphiploid and the lack of these amplicons in addition lines containing chromosomes 1-2E and 4-7E, were mapped on the group 3 chromosomes of wheat as well.

Three markers were located on non-homoeologous chromosomes in *Thinopyrum* relative to wheat: the w5-specific marker *TR759* on 4E, the w6-specific marker *c741435* on 4E and the w5-specific marker *c749645* could mapped on 5E and on 7E, too. These markers may indicate genome rearrangements in the E genome relative to wheat.

The chromosomal location of COS markers revealed a large-scale chromosome rearrangement and several intragenomic duplications in the *Thinopyrum elongatum* chromosomes (Table 1; Figs 4–6). Eight markers specific for the group 2 chromosomes of wheat detected a large-scale chromosome rearrangement involved in 2EL and 6ES. Markers *TR430* and *TR636*, specific for the pericentromeric region of wheat chromosomes 2BL and 2DL, indicated that a small centromeric chromatine present in the 6ES telosome is also involved in the 2ES/6EL rearrangement (it is important to note that *TR636* was also located in both 6ES and 6EL), while the markers *BE443185*, *TR146*, *BE495373*, *c744070*, *BE424990*, *2N* and *c746642*, specific for the interstitial and distal parts of the wheat group 2 long chromosome arm, detected these regions in the long arm of 2E and on the 6E. Small-scale duplications detected by single markers were also identified. The *c749645* loci specific for the long arm of w5 chromosomes showed 5EL/7ES duplication, respectively.

The location of the COS markers on the chromosome arms of *Thinopyrum* and wheat allowed wheat-*Thinopyrum* macrosyntenic relationships to be visualized at the chromosomal arm level. The marker content of the short and long arms of the w1 and w2 chromosomes were mapped on the homoeologous chromosome arms of the E genome. The same was observed for the short arm of group 6 chromosomes of wheat and, to a lesser extent, for the group 7 chromosomes.

However, the presence of markers specific for the short arm of a wheat chromosome on the long arm of an E chromosome or vice-versa may indicate intrachromosomal rearrangements in *Th*. *elongatum* relative to wheat, as detected for the short and long arms of w4, to some extent for w7 chromosomes, and for the long arms of w5 chromosomes.

## Discussion

While cytogenetic methods are valuable for genomic constitution verification of wheat-*Th*. *elongatum* interspecific hybrids, molecular markers are irreplaceable for fast, reliable and effective screening during introgression breeding programmes and for preferential identification of micro-introgressions.

In the present study, a 169 markers were selected for identification of chromosomes or chromosomal arms of *Th*. *elongatum* in wheat genetic background. Out of the 156 markers which produced amplicons, 60 (38.4%) were polymorphic between wheat and *Th*. *elongatum*. The transferability of COS markers was also investigated for other species [52–54]. In the tribe *Triticeae* Molnár et al. [40] found 94.3% transferability between wheat and *Aegilops* species containing U and M genomes in diploid or polyploid form. In the case of other *Aegilops* species containing S, U, M and D genomes, a similar level (>90%) of transferability was reported [36]. On the other hand, when Copete and Cabrera [55] investigated the utility of COS markers to detect the transfer of the P-genome chromosomes of *Agropyron cristatum* into wheat, a lower transferability of the COS markers (71.7% and 64.1% for the group 2 and group 6 markers, respectively) compared to *Aegilops* was reported. The higher percentage of transferability to *Aegilops* species compared with wheatgrasses can be attributed to the closer phylogenetic relationship of *Triticum* to *Aegilops*, which is known to have played an important role in the genomic evolution of wheat [56].

In the present work, 60 polymorphic markers were assigned to the chromosomes or chromosome arms of the E genome and forty-eight of these were assigned to a single chromosome of *Th*. *elongatum*. These easy-to-use gene-specific markers are suitable for use in prebreeding programmes for the preselection of wheat-*Th*. *elongatum* hybrid progenies and backcrossed genotypes for the presence of desirable E-genome chromosomes or chromosome arms. By combining these molecular markers with cytomolecular tools (FISH and GISH), the efficiency of chromosome-mediated gene transfer from *Thinopyrum elongatum* to wheat can be improved.

It has been reported that several QTLs and genes responsible for the agronomically useful traits of *Th*. *elongatum* have been assigned to the E-genome chromosomes. For example, several loci providing tolerance to salt stress have been mapped on chromosomes 3E, 4E and 7E [57]. A resistance gene against wheat streak mosaic virus (WSMV) was located on 1EL [9], while genes conferring resistance against leaf rust or stem rust were located on 3E (*Lr24*/*Sr24*), 6E (*Lr29*/*Sr26*) and 7EL (*Lr19*/*Sr25*) [1]. It was also reported that the 7E chromosome possesses high resistance to Fusarium head blight, so it is of great interest to breeders [58,59]. Although several markers have been designed to map these traits and used for marker-assisted selection [60–62], some of them are labourious and time-consuming to use or sometimes lose genetic linkage to the gene of interest. The COS marker technology offers an advantage for the targeted development of markers tightly linked to genes, as demonstrated by Burt and Nicholson [39], who developed markers to map the *Aegilops ventricosa*-derived *Pch1* eyespot resistance in wheat. In their work, knowledge about genome co-linearity between grass species was utilised for marker development.

In order to get a better insight into wheat-*Th*. *elongatum* macrosyntenic relationships, the source EST sequences of the polymorphic COS markers were mapped on the reference sequences of wheat chromosomes w1-w7. The present study generally showed close homologous relationships between the chromosome arms of wheat and *Th*. *elongatum*. However, this arm-level homology was perturbed in some loci. Two markers on the w5 long arm reflected homology with 4EL or 7EL and one marker on the w6 chromosomes showed homology with 4EL.

The present results for wheat-*Th*.*elongatum* macrosyntenic relationships agreed well with those observed by Zhou et al. [63] for the P-genome in the case of wheat—*A*. *cristatum* relationship. According to the 660K SNP array-based segregating genetic map for *A*. *cristatum* x *A*. *mongolicum*, the authors also found that the P genome of *Agropyron* is generally collinear with the wheat genomes, but several small-scale genome rearrangements exist throughout the P genome relative to wheat.

The present study showed that the wheat-*Th*. *elongatum* 6E disomic addition line contains a 6ES-2EL translocation. The perfect determination of translocation breakpoint was not possible by GISH, but the marker results indicates that it can be centromeric or close to the centromere in the 2ES arm as two markers specific for the short arm of w2 were also detected on 2ES and 6ES arms. Because of the wheat-*Th*. *elongatum* amphiploid contains a 6E chromosome with different hybridization pattern, it can be concluded that the translocation was evolved during the development of the addition lines through the backcrossing and selfing of the amphiploid. Further studies are needed to a more perfect characterisation of this translocation line.

Small-scale duplications of loci located on wheat group 5, 6 and 7 (7A and 7B) chromosomes were also detected in the E genome of *Th*. *elongatum*. The present results indicating the presence of small-scale duplications agreed well with those of a previous study made by Hu et al. [64], who also found several small-scale duplications (1E/2E, 1E/6E, 1E/4E, 2E/3E and 7E/4E) in the E genome of *Th*. *elongatum* when using EST-SSR markers. It is known that a considerable percentage of genes have been duplicated not only in wheat [65] and barley [66] but also in rice, sorghum and maize [67]. Small-scale duplications have also been found in wild relatives of wheat, such as *Aegilops* species with U and M genomes [40]. The present data support a recent model suggesting that a series of whole genome and segmental duplications, chromosome fusions, and translocations played an important role in the evolution of recent grass genomes [67]. In this study two or three chromosomes were involved in the interchromosomal duplication events. It has been suggested that several mechanisms play a role in gene duplication in the plant genomes, including transposable elements and/or ectopic recombination, as a part of double strand break repair, which may be involved in the formation of segmental or small-scale duplications in *Th*. *elongatum*. In wheat, for example CACTA transposons were found to move 140 non-syntenic genes [68].

In a recent gene enrichment analysis on chromosome 3B of wheat showed that a significant number of non-syntenic duplicated genes have GO annotations of programmed cell dead which is related to the defense strategies against abiotic stress and to disease resistance [69]. In this context, several resistance genes have been transferred from *Th*. *elongatum* to wheat (*Lr19/Sr25*; *Lr29/Sr26*; *Lr24/Sr24*) [1] and it can be hypothesised that the segmental duplication of genes may also be related to the evolution of disease resistance genes as a part of the adaptation to a stressful environment. However, a further high resolution genome analysis of *Th*. *elongatum* will be needed to obtain a deeper insight into the extent of duplicated genes and their relationship with the evolution of resistance genes.

The use of wheat-*Th*. *elongatum* ditelosomic lines allowed us to investigate wheat-*Thinopyrum* homology at the chromosome arm level. It was found that wheat short arm-specific markers were located on the short arms of the E genome chromosomes in the case of group 1, 2, 6 and 7 (7DS). The same homology was suggested for the long arms of groups 1 and 2 and one marker indicated homology between the long arms of 7AL and 7DL to 7EL. However, this arm-level homology was significantly disturbed in the case of 4E and 5E and also for 7E indicating that intrachromosomal rearrangements such as pericentric inversions may also have played a significant role in the karyotypic evolution of *Th*. *elongatum*.

## Conclusions

Perennial wheatgrass species are important gene reservoirs for wheat improvement as they contain new alleles and gene variants making it possible to cope with biotic and abiotic stresses in a changing environment. *Thinopyrum elongatum* has already been used in introgression breeding programmes to transfer agronomically important resistance genes into wheat. The conserved orthologous set markers assigned here to the chromosomes and chromosome arms of *Th*. *elongatum* promise to speed up the gene transfer by identifying the E-genome chromatin. Finally, the wheat-*Th*. *elongatum* macrosyntenic relationships established in this work will facilitate development of new markers specific for E genome in targeted regions and will contribute to the understanding of molecular processes related to polyploidization in the perennial wheatgrasses.

## Supporting information

S1 Fig Molecular cytogenetic analysis of the wheat-*Th*. *elongatum* addition lines. Presence and identity verification of wheat- *Th*. *elongatum* addition lines. A mitotic metaphase cell of the 1E disomic addition line after fluorescence *in situ* hybridization (FISH) (a) and genomic *in situ* hybridization (GISH) (b). A mitotic metaphase cell of the 4E disomic addition line after fluorescence *in situ* hybridization (FISH) (c) and genomic *in situ* hybridization (GISH) (d). A mitotic metaphase cell of the 5E disomic addition line after fluorescence *in situ* hybridization (FISH) (e) and genomic *in situ* hybridization (GISH) (f). In the GISH images the E genome was visualized in green, while in the FISH images the repetitive DNA probes pSc119.2, Afa family and pTa71 were visualized in green, red and yellow, respectively. Chromatin was nonspecifically stained with DAPI (blue). Scale bar = 10μm.
(TIF)

S2 Fig Molecular cytogenetic analysis of the wheat-*Th*. *elongatum* addition lines. Presence and identity verification of wheat- *Th*. *elongatum* addition lines. A mitotic metaphase cell of the 6E disomic addition line after fluorescence *in situ* hybridization (FISH) (a) and genomic *in situ* hybridization (GISH) (b). A mitotic metaphase cell of the 7E disomic addition line after fluorescence *in situ* hybridization (FISH) (c) and genomic *in situ* hybridization (GISH) (d). In the GISH images the E genome was visualized in green, while in the FISH images the repetitive DNA probes pSc119.2, Afa family and pTa71 were visualized in green, red and yellow, respectively. Chromatin was nonspecifically stained with DAPI (blue). Scale bar = 10μm.
(TIF)

S3 Fig Molecular cytogenetic analysis of the wheat-*Th*. *elongatum* addition lines. Presence and identity verification of wheat- *Th*. *elongatum* addition lines. A mitotic metaphase cell of the 1ES ditelosomic addition line after fluorescence in situ hybridization (FISH) (a) and genomic in situ hybridization (GISH) (b). A mitotic metaphase cell of the 1EL ditelosomic addition line after fluorescence in situ hybridization (FISH) (c) and genomic in situ hybridization (GISH) (d). A mitotic metaphase cell of the 2ES ditelosomic addition line after fluorescence in situ hybridization (FISH) (e) and genomic in situ hybridization (GISH) (f). A mitotic metaphase cell of the 2EL ditelosomic addition line after fluorescence in situ hybridization (FISH) (g) and genomic in situ hybridization (GISH) (h). In the GISH images the E genome was visualized in green, while in the FISH images the repetitive DNA probes pSc119.2, Afa family and pTa71 were visualized in green, red and yellow, respectively. Chromatin was nonspecifically stained with DAPI (blue). Scale bar = 10μm.
(TIF)

S4 Fig Molecular cytogenetic analysis of the wheat-*Th*. *elongatum* addition lines. Presence and identity verification of wheat- *Th*. *elongatum* addition lines. A mitotic metaphase cell of the 4ES ditelosomic addition line after fluorescence in situ hybridization (FISH) (a) and genomic in situ hybridization (GISH) (b). A mitotic metaphase cell of the 5ES ditelosomic addition line after fluorescence in situ hybridization (FISH) (c) and genomic in situ hybridization (GISH) (d). A mitotic metaphase cell of the 5EL ditelosomic addition line after fluorescence in situ hybridization (FISH) (e) and genomic in situ hybridization (GISH) (f). A mitotic metaphase cell of the 6ES ditelosomic addition line after fluorescence in situ hybridization (FISH) (g) and genomic in situ hybridization (GISH) (h). In the GISH images the E genome was visualized in green, while in the FISH images the repetitive DNA probes pSc119.2, Afa family and pTa71 were visualized in green, red and yellow, respectively. Chromatin was nonspecifically stained with DAPI (blue). Scale bar = 10μm.
(TIF)

S5 Fig Molecular cytogenetic analysis of the wheat-*Th*. *elongatum* addition lines. Presence and identity verification of wheat- *Th*. *elongatum* addition lines. A mitotic metaphase cell of the 7ES ditelosomic addition line after fluorescence in situ hybridization (FISH) (a) and genomic in situ hybridization (GISH) (b). A mitotic metaphase cell of the 7EL ditelosomic addition line after fluorescence in situ hybridization (FISH) (c) and genomic in situ hybridization (GISH) (d). In the GISH images the E genome was visualized in green, while in the FISH images the repetitive DNA probes pSc119.2, Afa family and pTa71 were visualized in green, red and yellow, respectively. Chromatin was nonspecifically stained with DAPI (blue). Scale bar = 10μm.
(TIF)

S1 Table The PCR conditions and the primer sequence’s of the COS marker analysis. (XLSX)

S2 Table The results of COS marker analysis. (XLSX)

S3 Table Results of BLASTn search for COS markers assigned to *Thinopyrum elongatum* chromosomes. (XLSX)
