# Supplementary figures and images for: Identification of COS markers specific for Thinopyrum elongatum chromosomes preliminary revealed high level of macrosyntenic relationship between the wheat and Th. elongatum genomes
Source: PLoS One. 2018 Dec 12;13(12):e0208840. doi: 10.1371/journal.pone.0208840 (PMC6291125; doi:10.1371/journal.pone.0208840)

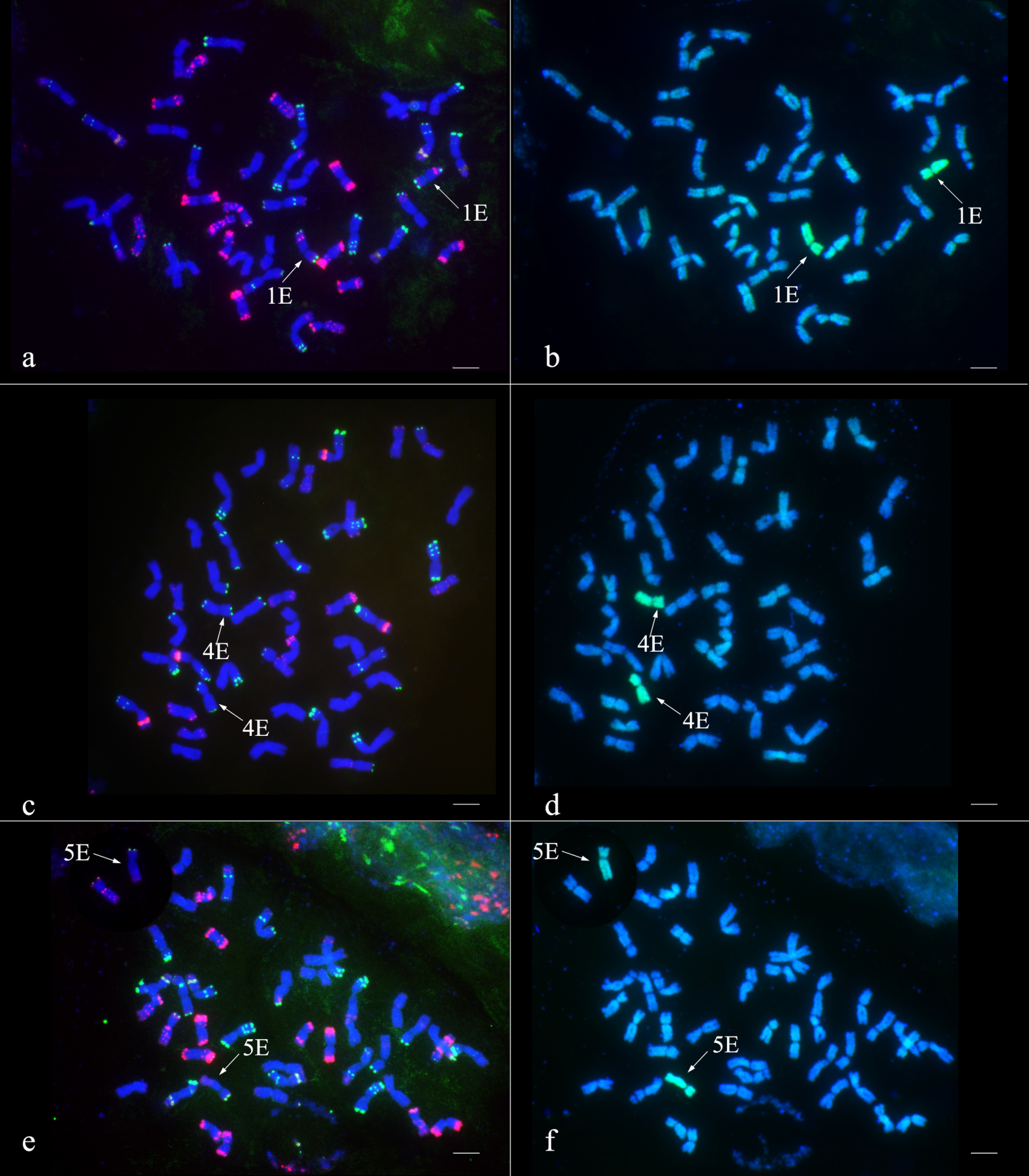

Supplement: S1 Fig — Presence and identity verification of wheat- Th. elongatum addition lines. A mitotic metaphase cell of the 1E disomic addition line after fluorescence in situ hybridization (FISH) (a) and genomic in situ hybridization (GISH) (b). A mitotic metaphase cell of the 4E disomic addition line after fluorescence in situ hybridization (FISH) (c) and genomic in situ hybridization (GISH) (d). A mitotic metaphase cell of the 5E disomic addition line after fluorescence in situ hybridization (FISH) (e) and genomic in situ hybridization (GISH) (f). In the GISH images the E genome was visualized in green, while in the FISH images the repetitive DNA probes pSc119.2, Afa family and pTa71 were visualized in green, red and yellow, respectively. Chromatin was nonspecifically stained with DAPI (blue). Scale bar = 10μm. (TIF) [file pone.0208840.s001.tif]

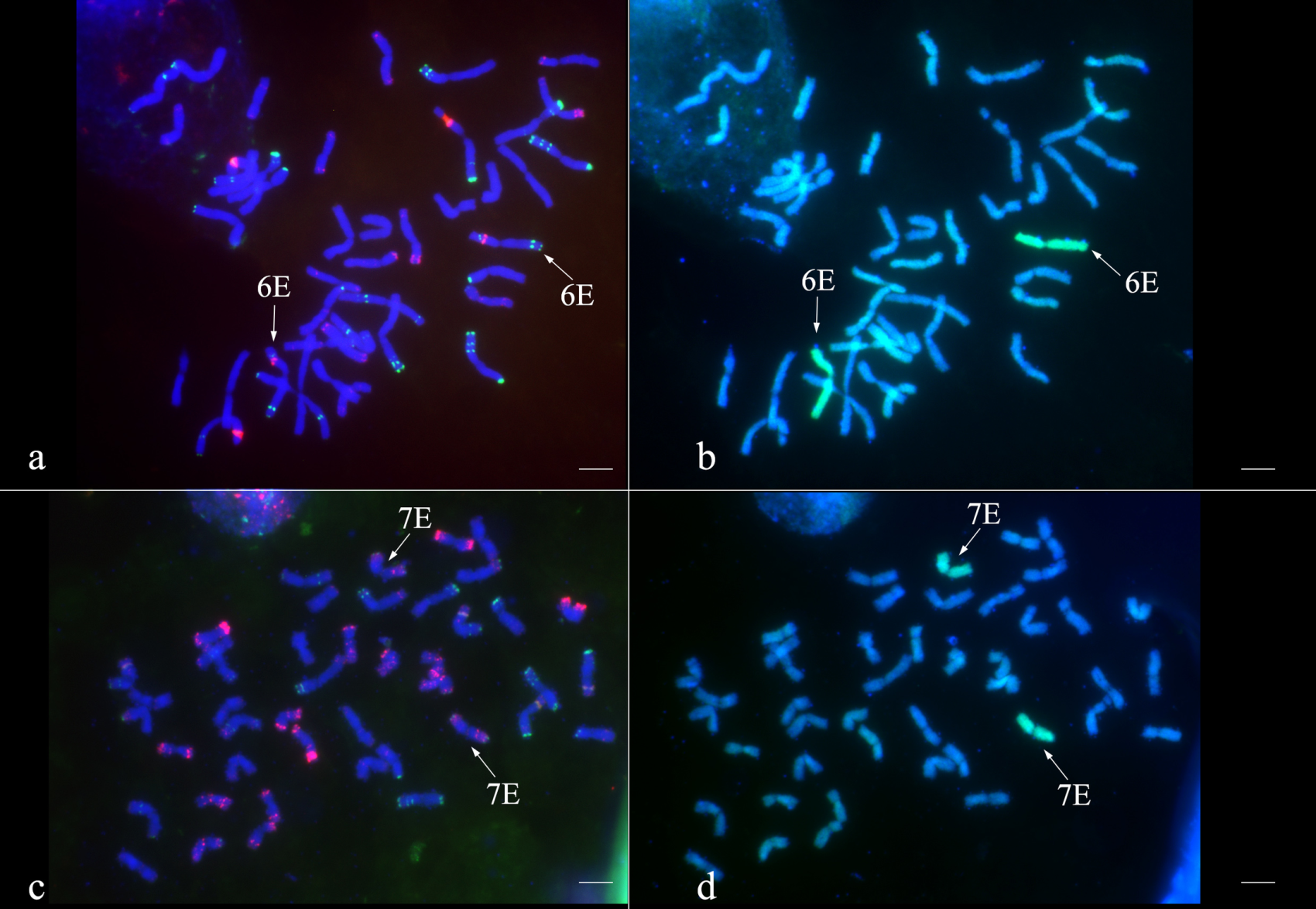

Supplement: S2 Fig — Presence and identity verification of wheat- Th. elongatum addition lines. A mitotic metaphase cell of the 6E disomic addition line after fluorescence in situ hybridization (FISH) (a) and genomic in situ hybridization (GISH) (b). A mitotic metaphase cell of the 7E disomic addition line after fluorescence in situ hybridization (FISH) (c) and genomic in situ hybridization (GISH) (d). In the GISH images the E genome was visualized in green, while in the FISH images the repetitive DNA probes pSc119.2, Afa family and pTa71 were visualized in green, red and yellow, respectively. Chromatin was nonspecifically stained with DAPI (blue). Scale bar = 10μm. (TIF) [file pone.0208840.s002.tif]

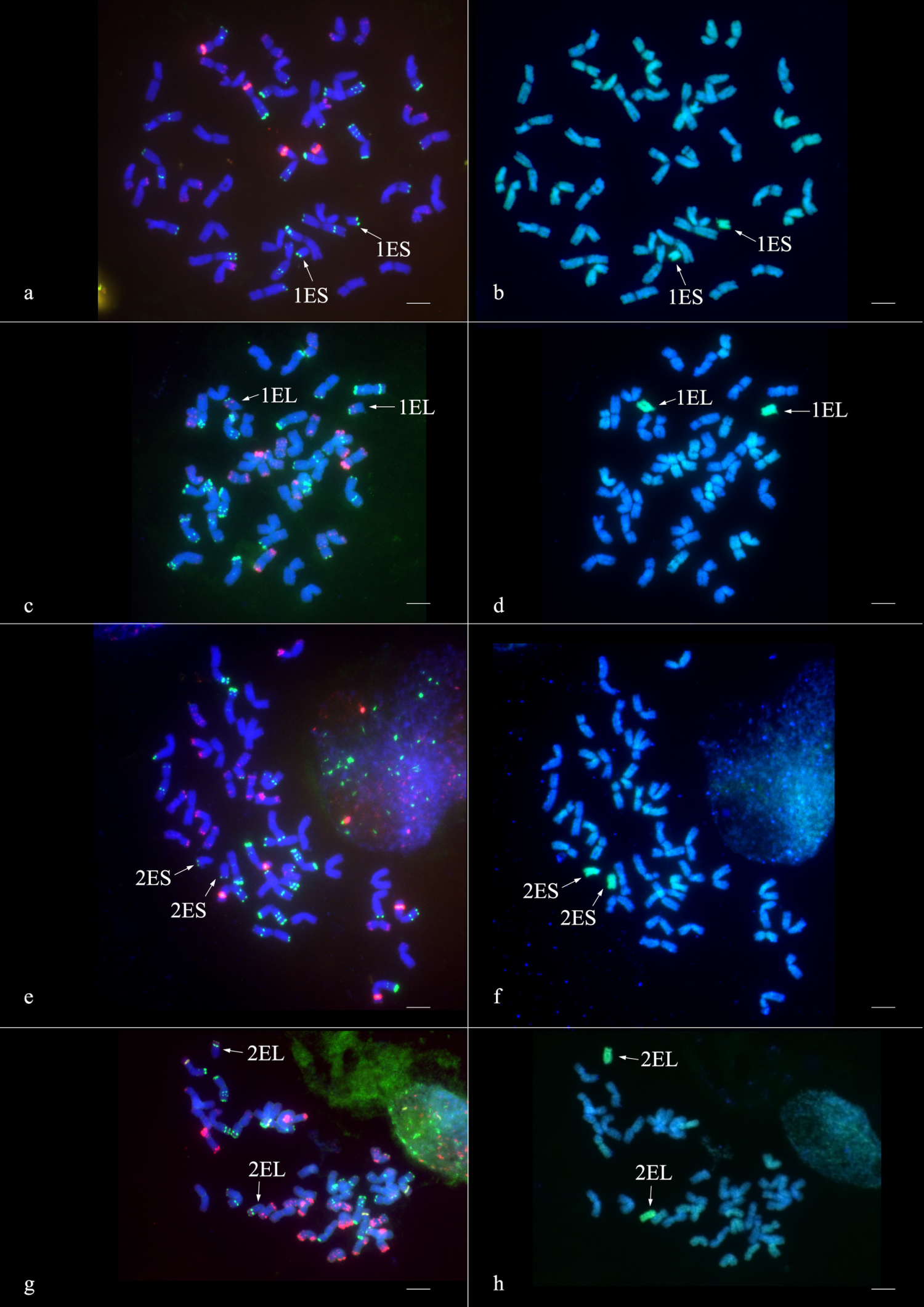

Supplement: S3 Fig — Presence and identity verification of wheat- Th. elongatum addition lines. A mitotic metaphase cell of the 1ES ditelosomic addition line after fluorescence in situ hybridization (FISH) (a) and genomic in situ hybridization (GISH) (b). A mitotic metaphase cell of the 1EL ditelosomic addition line after fluorescence in situ hybridization (FISH) (c) and genomic in situ hybridization (GISH) (d). A mitotic metaphase cell of the 2ES ditelosomic addition line after fluorescence in situ hybridization (FISH) (e) and genomic in situ hybridization (GISH) (f). A mitotic metaphase cell of the 2EL ditelosomic addition line after fluorescence in situ hybridization (FISH) (g) and genomic in situ hybridization (GISH) (h). In the GISH images the E genome was visualized in green, while in the FISH images the repetitive DNA probes pSc119.2, Afa family and pTa71 were visualized in green, red and yellow, respectively. Chromatin was nonspecifically stained with DAPI (blue). Scale bar = 10μm. (TIF) [file pone.0208840.s003.tif]

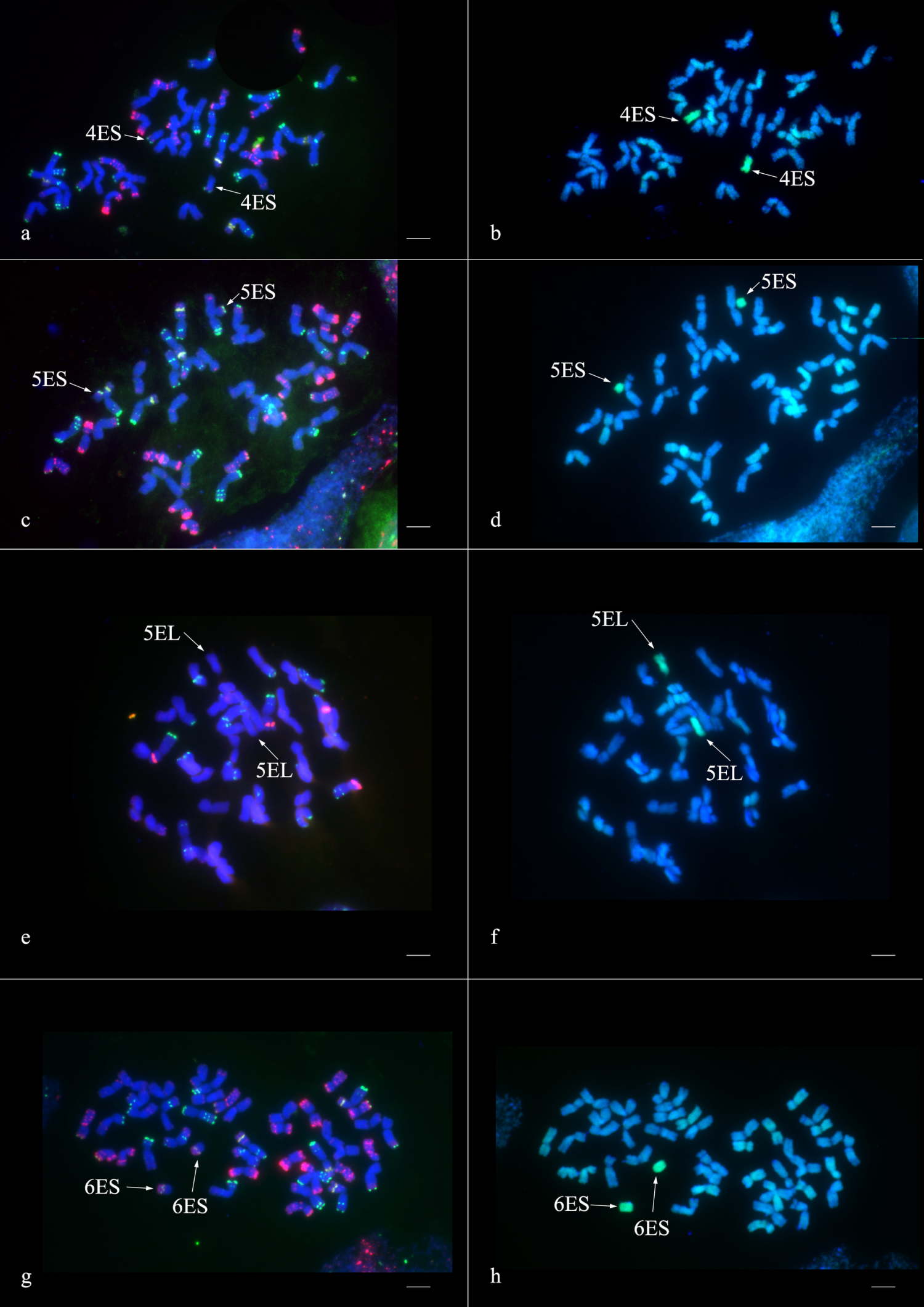

Supplement: S4 Fig — Presence and identity verification of wheat- Th. elongatum addition lines. A mitotic metaphase cell of the 4ES ditelosomic addition line after fluorescence in situ hybridization (FISH) (a) and genomic in situ hybridization (GISH) (b). A mitotic metaphase cell of the 5ES ditelosomic addition line after fluorescence in situ hybridization (FISH) (c) and genomic in situ hybridization (GISH) (d). A mitotic metaphase cell of the 5EL ditelosomic addition line after fluorescence in situ hybridization (FISH) (e) and genomic in situ hybridization (GISH) (f). A mitotic metaphase cell of the 6ES ditelosomic addition line after fluorescence in situ hybridization (FISH) (g) and genomic in situ hybridization (GISH) (h). In the GISH images the E genome was visualized in green, while in the FISH images the repetitive DNA probes pSc119.2, Afa family and pTa71 were visualized in green, red and yellow, respectively. Chromatin was nonspecifically stained with DAPI (blue). Scale bar = 10μm. (TIF) [file pone.0208840.s004.tif]

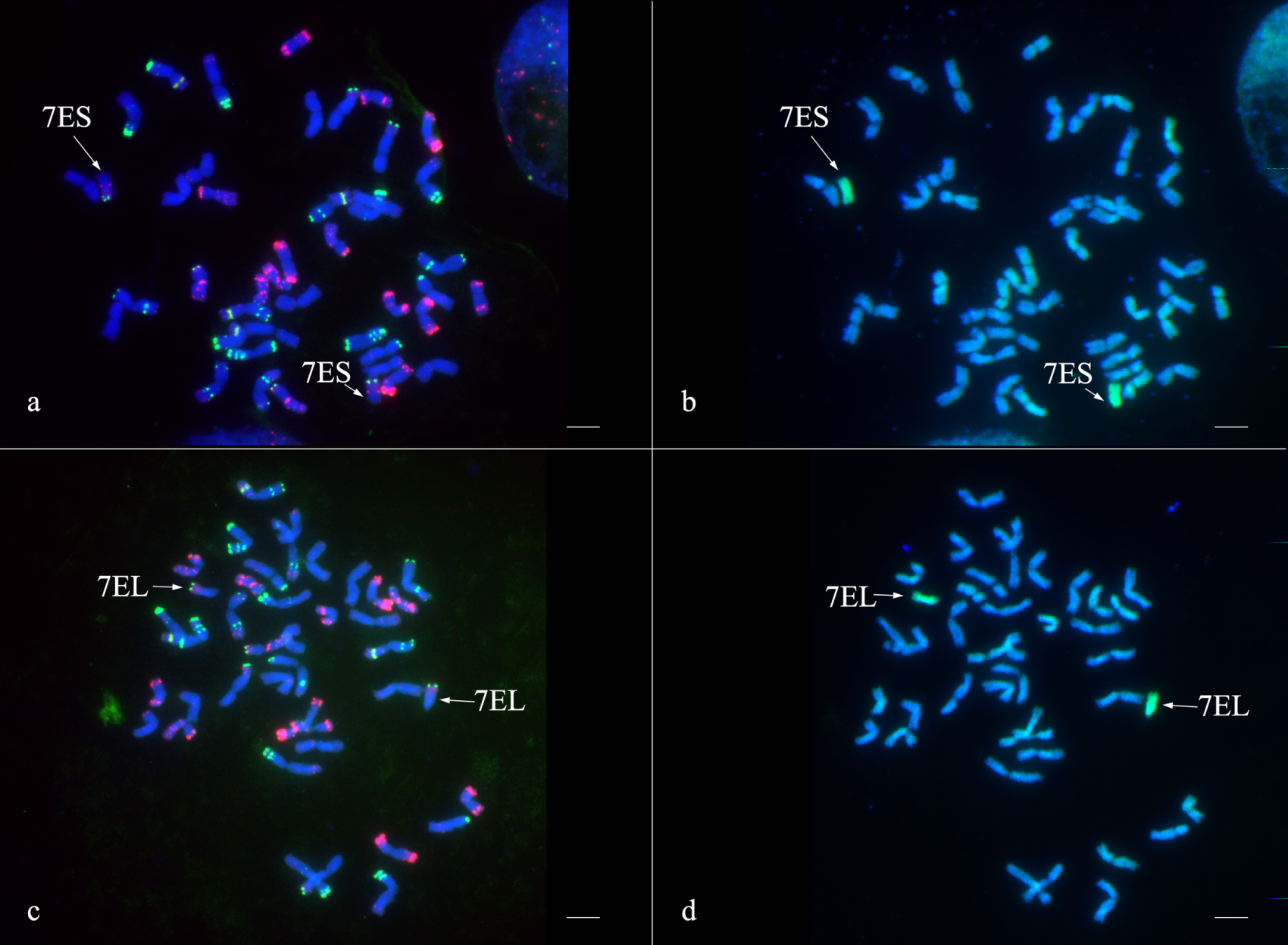

Supplement: S5 Fig — Presence and identity verification of wheat- Th. elongatum addition lines. A mitotic metaphase cell of the 7ES ditelosomic addition line after fluorescence in situ hybridization (FISH) (a) and genomic in situ hybridization (GISH) (b). A mitotic metaphase cell of the 7EL ditelosomic addition line after fluorescence in situ hybridization (FISH) (c) and genomic in situ hybridization (GISH) (d). In the GISH images the E genome was visualized in green, while in the FISH images the repetitive DNA probes pSc119.2, Afa family and pTa71 were visualized in green, red and yellow, respectively. Chromatin was nonspecifically stained with DAPI (blue). Scale bar = 10μm. (TIF) [file pone.0208840.s005.tif]
